# Interdependency Between Autophagy and Synaptic Vesicle Trafficking: Implications for Dopamine Release

**DOI:** 10.3389/fnmol.2018.00299

**Published:** 2018-08-21

**Authors:** Fiona Limanaqi, Francesca Biagioni, Stefano Gambardella, Larisa Ryskalin, Francesco Fornai

**Affiliations:** ^1^Human Anatomy, Department of Translational Research and New Technologies in Medicine and Surgery, University of Pisa, Pisa, Italy; ^2^IRCCS Neuromed, Pozzilli, Italy

**Keywords:** cell-clearing systems, mTOR, RabGTPase, SNARE, retromer, exosome, endosome, Munc13

## Abstract

Autophagy (ATG) and the Ubiquitin Proteasome (UP) are the main clearing systems of eukaryotic cells, in that being ultimately involved in degrading damaged and potentially harmful cytoplasmic substrates. Emerging evidence implicates that, in addition to their classic catalytic function in the cytosol, autophagy and the proteasome act as modulators of neurotransmission, inasmuch as they orchestrate degradation and turnover of synaptic vesicles (SVs) and related proteins. These findings are now defining a novel synaptic scenario, where clearing systems and secretory pathways may be considered as a single system, which senses alterations in quality and distribution (in time, amount and place) of both synaptic proteins and neurotransmitters. In line with this, in the present manuscript we focus on evidence showing that, a dysregulation of secretory and trafficking pathways is quite constant in the presence of an impairment of autophagy-lysosomal machinery, which eventually precipitates synaptic dysfunction. Such a dual effect appears not to be just incidental but it rather represents the natural evolution of archaic cell compartments. While discussing these issues, we pose a special emphasis on the role of autophagy upon dopamine (DA) neurotransmission, which is early affected in several neurological and psychiatric disorders. In detail, we discuss how autophagy is engaged not only in removing potentially dangerous proteins, which can interfere with the mechanisms of DA release, but also the fate of synaptic DA vesicles thus surveilling DA neurotransmission. These concepts contribute to shed light on early mechanisms underlying intersection of autophagy with DA-related synaptic disorders.

## Introduction

The intimate interplay between Autophagy (ATG), Ubiquitin Proteasome (UP) and neurotransmission has assumed increasing interest in the context of several neurodegenerative disorders such as Alzheimer’s disease (AD), Parkinson’s disease (PD) and Huntington’s disease (HD), which fit the definition of “synaptopathic proteinopathies.” A common hallmark underlying the physiopathology of parkinsonism is the early disruption of dopamine (DA) neurotransmission, which is also implicated in numerous neuropsychiatric disorders including schizophrenia and drug addiction (Belujon and Grace, 2017; Weinstein et al., 2017; Limanaqi et al., 2018). It is well established that in DA-related disorders the highly reactive nature of DA provides *per se* a basis for the high vulnerability of DA-containing neurons to oxidative stress-related damage, a context in which the proteolytic role of ATG and UP is crucial (Lazzeri et al., 2007; Pasquali et al., 2008). In support to this view, recent studies confirmed that genetic or pharmacologic inhibition of ATG in DA neurons fully reproduces PD and exacerbates the neurotoxic effects of the abused drug methamphetamine (Castino et al., 2008; Lin et al., 2012; Sato et al., 2018). Nonetheless, a novel scenario is emerging in which, beyond its well-established role in neurotoxicity-related mechanisms, ATG plays a key role in neurotransmitter release at either central or peripheral synapses (Hernandez et al., 2012; Binotti et al., 2015; Shen et al., 2015). In such a context, understanding the modulatory dynamics behind DA release is key, since DA represents a quintessential neurotransmitter implicated in regulating brain functions such as movement, cognition, attention, memory and reward (Sillitoe and Vogel, 2008), which are similarly affected in PD, schizophrenia and drug addiction. Therefore, studying the implication of ATG in early events underlying altered DA-neurotransmission, especially DA synaptic vesicles (SVs) turnover and DA release may offer insights on the physiopathology of DA-system disorders. In the light of these considerations, the present review article focuses on the major findings dealing with the role of ATG in modulating neurotransmission while posing a special emphasis on those related with DA release. In keeping with this, mTOR (mammalian Target of Rapamycin) which is a main upstream regulator of both ATG and UP (Zhao et al., 2015; Lenzi et al., 2016), has been widely implicated in synaptic plasticity and DA-related brain disorders (Lipton and Sahin, 2014; Ryskalin et al., 2018b). These findings may contribute to unravel novel neuroprotective strategies in “mTORopathies” specifically aimed to counteract synaptic toxicity.

## Autophagy and the Secretory Pathway

**Figure 1:**
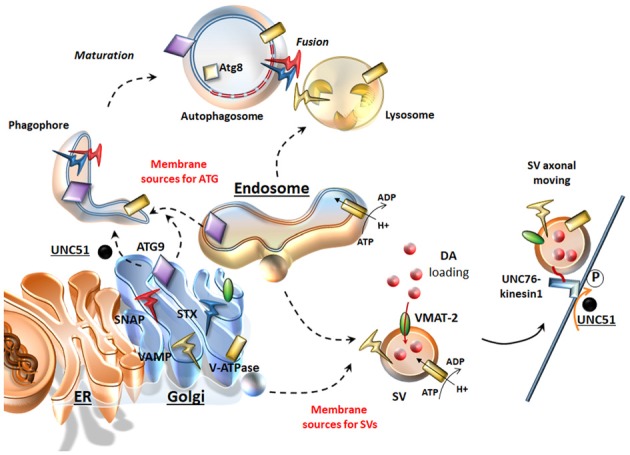
Similarities between the secretory pathway and autophagy (ATG). The Endoplasmic Reticulum (ER), Golgi and endosomes are sources for both ATG and synaptic vesicles (SVs). Once ATG is initiated upon UNC51/Atg1, ATG9 shuttles towards nascent ATG vacuoles where key proteins like Atg8/LC3 are recruited. From these same very same sources dopamine (DA)-SVs originate by membrane budding. In addition, these organelles provide proteins, which are key for both SVs and ATG. These include DA-SV specific proteins such as the vesicular monoamine transporter type-2 (VMAT-2; which allows DA storage), but also the V-ATPase (which is essential for intraluminal acidification of both ATG-lysosomes and SVs) and the Soluble NSF (N-ethylmaleimidesensitivefactor (SNARE) proteins SNAP, VAMP, Syntaxin (STX), which are essential for SV fusion and release but also for the maturation and homotypic fusion of ATG vacuoles. Similarly, UNC51/Atg1 is key not only to initiate and translocate ATG, but also to promote SVs translocation by phosphorylating the kinesin-1 adaptor UNC76.

Presynaptic neurotransmitter release is finely tuned by the secretory cycle of SVs, which includes key steps ranging from SV formation to SV’s protein sorting, loading of neurotransmitters within SVs, and, once at the active zone: SV docking, priming, and Ca^2+^-driven exocytotic release; this is eventually followed by SV endocytic recycling and/or degradation (Südhof, 2004; Rizzoli, 2014). Such a complex cycle entails dynamic interactions of multiple machineries of both the secretory-trafficking pathways and ATG, which are now starting to shape as a single system operating either in the cytosol (just as classic ATG machinery) or at the synapse (to tune neurotransmitter release and again acting as a protein-clearing system; Farhan et al., 2017; Vijayan and Verstreken, 2017). In line with this, a dysregulation of secretory pathways is quite constant in the presence of an impairment of ATG-lysosomal function and vice-versa (Shen et al., 2015). ATG intersection with secretory pathways is grounded on mutual interactions both at ultrastructural and molecular levels. In fact, several organelles of the secretory pathway provide membrane sources for both ATG vacuoles and SVs (Hannah et al., 1999; Longatti and Tooze, 2012; Rizzoli, 2014; Bento et al., 2016; Figure 1). In general, SVs originate by budding from these sources, which correspond to those where ATG initiation takes place. In fact, DA-SVs are represented primarily by Golgi-derived small SVs (SSVs) and large dense core vesicles (LDCVs), as well as tubule-vesicular structures resembling smooth ER stores (Nirenberg et al., 1996). Such a remarkable overlapping in vesicle sources for ATG and SVs may be regulated by similar molecular complexes. For instance, ATG initiation is promoted by UNC51-like kinase 1/2 complex (the *C. Elegans* homolog of the mammalian ULK1/2). Remarkably, a loss of UNC51 apart from impairing ATG initiation also dampens the axonal transport of SVs. In fact, a loss of UNC51 impairs the activity of UNC76 kinesin adaptor protein (Toda et al., 2008), which moves SVs towards the active zone by binding to the SV protein synaptotagmin-1 (Figure 1). Upon induction of ATG, ULK1/2 complex requires shuttling of the key trans-membrane protein Atg9 between membrane sources towards nascent ATG vacuoles (Takahashi et al., 2011; Yamamoto et al., 2012; Puri et al., 2013). In this way, Atg9 plays an important role in recycling membranes from ER, endosomal and Golgi sources and likely, even in recruiting endosomes and lysosomes by facilitating vesicular fusion (Figure 1). Beyond ATG compartments, occurrence of Atg9 within axons is required for synapse development (Stavoe et al., 2016), thus confirming a dual role for ATG proteins at the synapse to move diverse vesicular compartments within a highly coordinated secretory network. The correct localization/removal of membrane-associated proteins at both vacuolar organelles and plasma membrane active zone widely depends on endosome trafficking and sorting mechanisms, where ATG is deeply involved. In keeping with this, there are sets of specific evolutionary conserved multitasking protein families such as Rabs, G coupled Ras-related proteins in brain (Rab GTPases) and Soluble NSF (N-ethylmaleimide-sensitive factor (SNAREs, attachment protein receptor), which regulate all membrane-bound intracellular trafficking pathways (Moreau et al., 2011; Zhen and Stenmark, 2015). These proteins exert a parallel regulation of SV-cycle and ATG-lysosomal function (Figure 1).

### Autophagy and the Endocytic SV Trafficking Pathway

**Figure 2:**
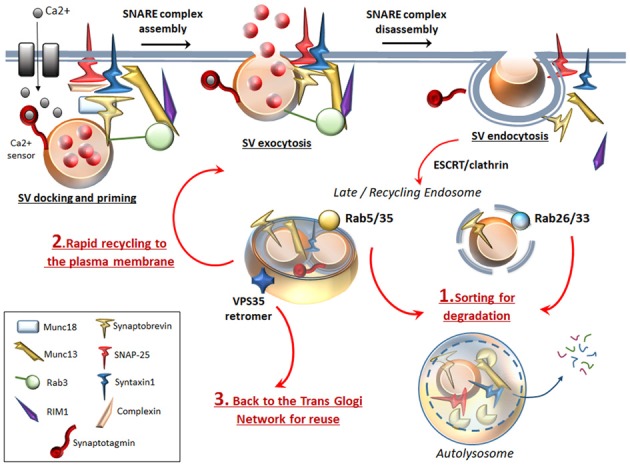
ATG surveils neurotransmission at the active zone. To complete SVs docking, priming and fusion, the SNARE proteins Synaptobrevin (VAMP), SNAP-25 and STX, require tethering proteins such as Munc18, Munc13 and the specialist proteins complexin, synaptotagmin. Complexin binds to partially assembled SNARE complexes during priming, and serves as an essential adaptor that enables synaptotagmin to sense intracellular Ca^2+^ increase. Munc13–1 forms a ternary complex with Rab3 and Rab3-interacting molecule (RIM1), which favors docking and priming and eventually exocytosis by opening the Munc18-mediated “closed” conformation of STX. Once exocytosis occurs, SNARE complex disassembles to allow endocytosis of SVs and associated proteins. Specific Rabs (Rab 5, 26, 33, 35) sort endocytosed SVs for ATG degradation (1). Rab26 and Rab33 reside specifically on SVs and target them directly for ATG-lysosomal degradation, while endosomal Rab5 and Rab35 sort SVs for ATG-lysosomal degradation via fusion with an endosomal intermediate. In this way, ATG degrades SNAP-25, synaptobrevin, Munc13 and whole SVs. This may be key to limit the potentiated neurotransmitter release, which would occur if SVs and associated proteins were rapidly recycled to the plasma membrane to promote a further round of exocytosis (2). Essential endocytosed components can also be targeted by the VPS35 retromer protein, which retrieves and traffics them back to the TGN for reuse (3).

Following neurotransmitter release, SVs recovered through endocytosis may differently invade presynaptic compartments: (i) Endocytosed SVs are locally recycled in order to replenish the ready-releasable pools for a further round of exo/endocytosis. (ii) Endocytosed SVs can be targeted by the retromer complex, which recycles SV-components by trafficking them back to the Trans Golgi Network for reuse (Inoshita et al., 2017; Vazquez-Sanchez et al., 2018). (iii) Endocytosed SVs can undergo ATG-lysosomal degradation (Frampton et al., 2012; Maday and Holzbaur, 2012; Fernandes et al., 2014; Binotti et al., 2015; Wang et al., 2015; Sheehan et al., 2016; Soukup et al., 2016; Okerlund et al., 2017). Main mechanisms that sort SV-cargoes for ATG-lysosomal degradation are the clathrin-dependent pathway (Ravikumar et al., 2010) and the endosomal sorting complex required for transport (ESCRT, Sheehan et al., 2016). In addition to the ESCRT pathway, several Rabs and specific SV-cycle proteins such as Endophilin-A represent an essential sorting mechanism to direct endocytosed SVs for recycling or for ATG degradation (Fernandes et al., 2014; Binotti et al., 2016; Sheehan et al., 2016; Soukup et al., 2016). In detail, among endosomal Rabs (Rab4, Rab5, Rab10, Rab11b, Rab14 and Rab35) which co-exist on SVs, Rab5 and Rab35 drive sorting of SVs for ATG-lysosomal degradation (Sheehan et al., 2016). Interestingly, two additional Rabs (Rab26 and Rab33a) are bound solely with SVs and ATG vacuoles, which indicate the uniqueness of SVs and ATG vacuoles in their molecular structure and function, while it poses a potential role for these Rabs to target SVs selectively for ATG degradation (Figure 2; Binotti et al., 2015). Dysfunctional Endophilin-A due to impaired phosphorylation by the LRRK2 kinase, disrupts both ATG and SV-cycle at the *Drosophila* neuromuscular junction (NMJ, Soukup et al., 2016). Since both LRRK2 and Endophilin-A are genetically linked to PD, these findings suggest a plausible molecular mechanism linking alterations of ATG and SV cycle in DA synapses. Mutated LRRK2 also associates with enhanced glutamate release and increased density of SV-associated proteins, which suggests that ATG may be involved in glutamate neurotransmission, though this has not been directly investigated yet (Beccano-Kelly et al., 2014). Again, dysfunctional or mutated Rab4, Rab5 and Rab11 impair both SV recycling and ATG compartments (Stenmark, 2009; Zhen and Stenmark, 2015; Binotti et al., 2016). In fact, Rab4, Rab5, Rab10 and Rab11 promote early steps of ATG such as phagophore formation and autophagosome maturation (Szatmári and Sass, 2014; Szatmári et al., 2014; Palmisano et al., 2017). It is worth of noting that, Rab5 and Rab11 are mandatory for appropriate trafficking and recycling of the DAT to the plasma membrane (Loder and Melikian, 2003; Furman et al., 2009; Hong and Amara, 2013), which remarks quite impressively the similarities between ATG and DA neurotransmission.

### Autophagy and the Exocytotic SV Pathway

Rabs bound to SVs also include the exocytotic Rab3 and Rab27B families, which tune SV-docking and -exocytosis (Pavlos et al., 2010; Binotti et al., 2016). Rab3GAP complex (Rab3GTP activating protein) is key to regulate neurotransmitter release by hydrolyzing GTP bound to Rab3 (Sakane et al., 2006). Remarkably, RabGAP1 and RabGAP2, which are the two components of Rab3GAP, modulate autophagosome biogenesis (Spang et al., 2014). In addition to Rabs, docking and priming of SVs to the active zone requires the SNARE proteins synaptobrevin/VAMP, SNAP-25 and Syntaxin-1 (STX). To complete SVs fusion, these latter require many other tethering proteins among which Munc18–1, Munc13–1, snapin, complexin, synaptotagmin and Bassoon (Südhof, 2013; Waites et al., 2013; Rizzoli, 2014). All these proteins collaboratively operate within a highly coordinated network to dynamically shape the active zone and finely tune SV’s exo/endocytosis (Figure 2). Nonetheless, these very same factors are also bound to ATG. Dysfunctional exo/endocytotic SNAREs have been found to negatively affect ATG function (Nair et al., 2011; Wang et al., 2016). Again, deletion of either synaptobrevin or snapin leads to aberrant endosomal trafficking and impaired ATG-lysosomal fusion (Cai et al., 2010; Haberman et al., 2012). Interestingly, synaptotagmin-11 was recently shown to be involved in ATG-lysosome machinery regulation in DA neurons (Bento et al., 2016). Again, ATG-lysosomal function is also sensitive to mutations of soluble NSF-attachment protein alpha (αSNAP), which together with NSF enzyme release single SNAREs from their complex in order to be recycled for subsequent rounds of SVs fusion (Ishihara et al., 2001; Abada et al., 2017). Interestingly, Bassoon, which is a scaffolding protein involved in organizing the active zone, was found to inhibit ATG-mediated synaptic degradation (Okerlund et al., 2017). On the other hand, ATG degrades several SNARE proteins, which participate in SVs priming and exocytosis (Uytterhoeven et al., 2015; Sheehan et al., 2016; Figure 2). It is worth mentioning that Hsc70, which regulates ATG at the *Drosophila* NMJ (Uytterhoeven et al., 2015), is also key in DA neurons to regulate the SV-sorting and activity of DA by interacting with both vesicular monoamine transporter type-2 (VMAT-2) and tyrosine hydroxylase (TH), the rate limiting enzyme for DA synthesis; Parra et al., 2016), which calls for further studies to unravel the intersection with ATG. Nonetheless, the present findings strongly suggest that the rate of presynaptic ATG-mediated protein degradation is key to determine the probability of neurotransmitter release.

### Autophagy and SV’s Retromer Sorting

Recent findings revealed that the retromer machinery in cooperation with Rab5 and Rab11 is key for DA neurotransmission in the light of its role to sort and retrieve a subset of endocytosed SVs away from the degradative pathway back to the TGN for reuse (Inoshita et al., 2017). DA neurons are susceptible to retromer perturbations, since VPS35 mutations lead to PD while VPS35 overexpression rescues PD phenotype (Linhart et al., 2014; Gambardella et al., 2016). Noteworthy, ATG induction and progression in DA neurons is tightly bound to the retromer function, which guarantees the correct trafficking and recycling of both Atg9 and Lamp2 lysosomal protein (Zavodszky et al., 2014; Tang et al., 2015). In fact, mutations of VPS35 cause endosomes perturbations, dysfunctional ATG (Zavodszky et al., 2014) and aberrant lysosomes, while producing altered DA outflow, dystrophic DA neurites/axons along with impaired motor behavior (Tang et al., 2015). Another key role of VPS35 in DA neurons is the sorting of VMAT-2 and DAT (Wu et al., 2016, 2017). In DA cell bodies and nerve terminals, the depletion of VPS35 disrupts VMAT-2 and DAT recycling to SVs and plasma membrane respectively, suggesting that retrograde trafficking is essential for DA storage and activity (Wu et al., 2016, 2017). Another cross-talk point between ATG and endosomal/retromer sorting pathways is related to intraluminal vesicle formation. Both Atg9 as well as the ESCRT and retromer machineries are required for formation, maturation and compartmentalized acidification of endosomal and ATG-lysosomal vacuoles (Rusten and Stenmark, 2009; Bader et al., 2015; Follett et al., 2017). Preservation of low vesicular pH is crucial not only for ATG function, but also for SV maturation, neurotransmitter loading into SVs and exocytotic release (Marshansky and Futai, 2008). Nonetheless, the retromer and its binding partner Wiskott Aldrich Syndrome protein and scar homolog (WASH) are also involved in the endosomal retrieval of the vesicular proton pump V-ATPase (Carnell et al., 2011), which maintains pH gradients within both SVs and ATG-lysosomal organelles (Rost et al., 2015). This suggests that a common phylogeny underlies the commonalities between secretory and degradative pathways. In line with this, both lysosomes and SVs may derive from early-endosomes (Newell-Litwa et al., 2009; Figure 1). Biogenesis of lysosome-related organelles complex (BLOC-1), may correspond to such an ancestral structure since it regulates maturation and trafficking of both SVs and ATG-lysosomes (Wang et al., 2017).

## Autophagy and DA Release

A role for lysosomal degradation in regulating SV turnover at nerve terminals was suggested since the early ‘70s (Holtzman et al., 1971). However, it was not until 2012 that a direct role for ATG machinery in modulating vesicular DA release was demonstrated (Hernandez et al., 2012). These authors generated mice lacking ATG7 specifically within DA neurons by expressing Cre recombinase under the control of the DA transporter DAT (Atg7-DAT-Cre mice). Measurement of DA release in the striatum was performed along with ultrastructural analyses of ATG, specifically within striatal axons and nerve terminals. The amplitude of DA signal evoked by electrical stimulation was 54% greater in Atg7-DAT-cre mice than in DAT-cre mice. On the other hand, inhibition of mTOR (and ATG activation) by rapamycin significantly decreases DA release of about 25% (corresponding quite precisely to a 25% reduction in the density of DA-SVs) only in DAT-cre mice and to a lesser extent in wild type mice. These findings suggest that ATG blunts DA transmission. Confocal microscopy, western blotting and electron microscopy analyses confirmed that ATG induction (as measured by an increase of LC3II particles along with ATG vacuoles) occurring locally within axons, reduces DA release via sequestration of presynaptic DA-SVs (Hernandez et al., 2012). Clear lumen ATG vacuoles fusing with endosomal vesicles were also observed which is in line with recent studies suggesting ATG-mediated degradation of endocytosed SVs occurring even in non-DA neurons (Binotti et al., 2015; Sheehan et al., 2016).

## Autophagy, Aggregation-Prone Proteins and Dopamine Release

The fine coordination between endosomal, retromer and ATG pathways is key for DA neurotransmission since it is involved in DA release, as well as membrane and vesicle re-uptake of DA. In this way, also DA toxicity is prevented, since DA-derived oxidative species can modify endogenous presynaptic chaperone proteins. This is the case of PD-related proteins, such as α-synuclein (α-syn), Parkin and LRRK2, which co-chaperon SVs’ trafficking and DA release by interacting physically with Rabs, SNAREs and Endophilin-A (Burré et al., 2010; Cao et al., 2014; Soukup et al., 2016; Shi et al., 2017; Ryskalin et al., 2018a). All these presynaptic proteins represent substrates for ATG-lysosomal degradation. As postulated by the “unconventional protein secretion” theory, organelles of the ATG and endo-lysosomal pathways may release these proteins thus mediating their inter-neuronal spreading via exosomes (Zhang and Schekman, 2013). In fact, just like SVs, the ATG-endo-lysosomal system can undergo exocytosis following intracellular Ca^2+^ increase. In these terms, this appears as a natural mechanism that neurons have conserved to communicate with each other and share essential cell constituents within a common environment. When dysfunctions in either ATG-endo-lysosomal pathways occur, indigested proteins are spread to neighbor cells (Lee et al., 2013), just in the same “uncontrolled” manner as DA may be powerfully released at the synaptic cleft to produce abnormal stimulation of post-synaptic DA receptors or even intra-neuronal spreading of reactive species (Jakel and Maragos, 2000). Thus, ATG-dependent modulation of vesicular DA trafficking and amount of released DA may potentially contribute to those mechanisms driving post-synaptic plasticity, such as long term-potentiation and -depression, which are directly related to neuronal and behavioral phenotypes. In detail, an abnormal stimulation of post-synaptic D1 DA receptors leads to series of non-canonical metabolic changes, which translate into activation of NMDA and AMPA glutamate receptors. These events potentiate glutamate release and Ca^2+^ entry within post-synaptic neurons thus promoting glutamate excitotoxicity. In this way, freely diffusible DA-derived free radicals together with glutamate-derived radical species synergize to produce detrimental effects in post-synaptic non-DA neurons. Here, the ATG pathway plays an equally crucial role by degrading AMPA receptors (Shehata et al., 2012) and by preventing glutamate-derived excitotoxic insult (Kulbe et al., 2014).

## Concluding Remarks

In the light of the tight interconnection of ATG with the secretory pathway, the findings revised in the present manuscript suggest that an impairment of ATG at the synapse may occur early, in parallel with SV cycle-related alterations. The hub linking ATG and DA release may be the tight interplay between ATG and innumerous proteins of the secretory pathway, which regulate both SV-cycle and ATG. This is supported by evidence showing that, mutations or dysfunctions of Rabs, SNAREs and Endophilin-A, which directly affect ATG, associate with several DA-synaptic disorders ranging from PD to drug addiction and schizophrenia (Drouet and Lesage, 2014; Katrancha and Koleske, 2015; Vanderwerf et al., 2015; Shi et al., 2017). On the other hand, ATG induction via mTOR or GSK3β ameliorates early psychomotor and cognitive behavioral alterations by rescuing neurotransmission defects in these same disorders (Schneider et al., 2016; Huang et al., 2018; Kara et al., 2018; Masini et al., 2018). In such a tightly interconnected mechanism, it is mandatory to further investigate the UP, which is also modulated by mTOR and co-localizes with ATG (Lenzi et al., 2016).

## Author Contributions

FL and FB wrote the article and made the artwork. SG contributed to important intellectual content. LR contributed to the literature review and artwork. FF: coordinator of the article. He participated in drafting the article. He participated in critically revising the article.

## Conflict of Interest Statement

The authors declare that the research was conducted in the absence of any commercial or financial relationships that could be construed as a potential conflict of interest.

## References

[B1] Abada A. Levin-Zaidman S. Porat Z. Dadosh T. Elazar Z. (2017). SNARE priming is essential for maturation of autophagosomes but not for their formation. Proc. Natl. Acad. Sci. U S A 114, 12749–12754. 10.1073/pnas.1705572114 29138318 PMC5715740

[B2] Bader C. A. Shandala T. Ng Y. S. Johnson I. R. D. Brooks D. A. (2015). Atg9 is required for intraluminal vesicles in amphisomes and autolysosomes. Biol. Open 4, 1345–1355. 10.1242/bio.013979 26353861 PMC4728360

[B3] Beccano-Kelly D. A. Kuhlmann N. Tatarnikov I. Volta M. Munsie L. N. Chou P.. (2014). Synaptic function is modulated by LRRK2 and glutamate release is increased in cortical neurons of G2019S LRRK2 knock-in mice. Front. Cell. Neurosci. 8:301. 10.3389/fncel.2014.00301 25309331 PMC4176085

[B4] Belujon P. Grace A. A. (2017). Dopamine system dysregulation in major depressive disorders. Int. J. Neuropsychopharmacol. 20, 1036–1046. 10.1093/ijnp/pyx056 29106542 PMC5716179

[B5] Bento C. F. Renna M. Ghislat G. Puri C. Ashkenazi A. Vicinanza M.. (2016). Mammalian autophagy: how does it work? Annu. Rev. Biochem. 85, 685–713. 10.1146/annurev-biochem-060815-014556 26865532

[B6] Binotti B. Jahn R. Chua J. J. (2016). Functions of rab proteins at presynaptic sites. Cells 5:E7. 10.3390/cells5010007 26861397 PMC4810092

[B7] Binotti B. Pavlos N. J. Riedel D. Wenzel D. Vorbrüggen G. Schalk A. M.. (2015). The GTPase Rab26 links synaptic vesicles to the autophagy pathway. Elife 4:e05597. 10.7554/eLife.05597 25643395 PMC4337689

[B8] Burré J. Sharma M. Tsetsenis T. Buchman V. Etherton M. R. Südhof T. C. (2010). α-synuclein promotes SNARE-complex assembly *in vivo* and *in vitro*. Science 329, 1663–1667. 10.1126/science.1195227 20798282 PMC3235365

[B9] Cai Q. Lu L. Tian J. H. Zhu Y. B. Qiao H. Sheng Z. H. (2010). Snapin-regulated late endosomal transport is critical for efficient autophagy-lysosomal function in neurons. Neuron 68, 73–86. 10.1016/j.neuron.2010.09.022 20920792 PMC2953270

[B10] Cao M. Milosevic I. Giovedi S. De Camilli P. (2014). Upregulation of Parkin in endophilin mutant mice. J. Neurosci. 34, 16544–16549. 10.1523/JNEUROSCI.1710-14.2014 25471590 PMC4252559

[B11] Carnell M. Zech T. Calaminus S. D. Ura S. Hagedorn M. Johnston S. A.. (2011). Actin polymerization driven by WASH causes V-ATPase retrieval and vesicle neutralization before exocytosis. J. Cell Biol. 193, 831–839. 10.1083/jcb.201009119 21606208 PMC3105540

[B12] Castino R. Lazzeri G. Lenzi P. Bellio N. Follo C. Ferrucci M.. (2008). Suppression of autophagy precipitates neuronal cell death following low doses of methamphetamine. J. Neurochem. 106, 1426–1439. 10.1111/j.1471-4159.2008.05488.x 18489716

[B13] Drouet V. Lesage S. (2014). Synaptojanin 1 mutation in Parkinson’s disease brings further insight into the neuropathological mechanisms. Biomed Res. Int. 2014:289728. 10.1155/2014/289728 25302295 PMC4181773

[B14] Farhan H. Kundu M. Ferro-Novick S. (2017). The link between autophagy and secretion: a story of multitasking proteins. Mol. Biol. Cell 28, 1161–1164. 10.1091/mbc.E16-11-0762 28468940 PMC5415012

[B15] Fernandes A. C. Uytterhoeven V. Kuenen S. Wang Y. C. Slabbaert J. R. Swerts J.. (2014). Reduced synaptic vesicle protein degradation at lysosomes curbs TBC1D24/sky-induced neurodegeneration. J. Cell Biol. 207, 453–462. 10.1083/jcb.201406026 25422373 PMC4242831

[B16] Follett J. Bugarcic A. Collins B. M. Teasdale R. D. (2017). Retromer’s role in endosomal trafficking and impaired function in neurodegenerative diseases. Curr. Protein Pept. Sci. 18, 687–701. 10.2174/1389203717666160311121246 26965691

[B17] Frampton J. P. Guo C. Pierchala B. A. (2012). Expression of axonal protein degradation machinery in sympathetic neurons is regulated by nerve growth factor. J. Neurosci. Res. 90, 1533–1546. 10.1002/jnr.23041 22411744 PMC3523670

[B18] Furman C. A. Lo C. B. Stokes S. Esteban J. A. Gnegy M. E. (2009). Rab 11 regulates constitutive dopamine transporter trafficking and function in N2A neuroblastoma cells. Neurosci. Lett. 463, 78–81. 10.1016/j.neulet.2009.07.049 19631257 PMC2754726

[B19] Gambardella S. Biagioni F. Ferese R. Busceti C. L. Frati A. Novelli G.. (2016). Vacuolar protein sorting genes in Parkinson’s disease: a re-appraisal of mutations detection rate and neurobiology of disease. Front. Neurosci. 10:532. 10.3389/fnins.2016.00532 27932943 PMC5121230

[B20] Haberman A. Williamson W. R. Epstein D. Wang D. Rina S. Meinertzhagen I. A.. (2012). The synaptic vesicle SNARE neuronal Synaptobrevin promotes endolysosomal degradation and prevents neurodegeneration. J. Cell Biol. 196, 261–276. 10.1083/jcb.201108088 22270918 PMC3265959

[B21] Hannah M. J. Schmidt A. A. Huttner W. B. (1999). Synaptic vesicle biogenesis. Annu. Rev. Cell Dev. Biol. 15, 733–798. 10.1146/annurev.cellbio.15.1.733 10611977

[B22] Hernandez D. Torres C. A. Setlik W. Cebrián C. Mosharov E. V. Tang G.. (2012). Regulation of presynaptic neurotransmission by macroautophagy. Neuron 74, 277–284. 10.1016/j.neuron.2012.02.020 22542182 PMC3578406

[B23] Holtzman E. Freeman A. R. Kashner L. A. (1971). Stimulation-dependent alterations in peroxidase uptake at lobster neuromuscular junctions. Science 173, 733–736. 10.1126/science.173.3998.733 4327989

[B24] Hong W. C. Amara S. G. (2013). Differential targeting of the dopamine transporter to recycling or degradative pathways during amphetamine- or PKC-regulated endocytosis in dopamine neurons. FASEB J. 27, 2995–3007. 10.1096/fj.12-218727 23612789 PMC3714572

[B25] Huang S. H. Wu W. R. Lee L. M. Huang P. R. Chen J. C. (2018). mTOR signaling in the nucleus accumbens mediates behavioral sensitization to methamphetamine. Prog. Neuropsychopharmacol. Biol. Psychiatry 86, 331–339. 10.1016/j.pnpbp.2018.03.017 29574227

[B26] Inoshita T. Arano T. Hosaka Y. Meng H. Umezaki Y. Kosugi S.. (2017). Vps35 in cooperation with LRRK2 regulates synaptic vesicle endocytosis through the endosomal pathway in *Drosophila*. Hum. Mol. Genet. 26, 2933–2948. 10.1093/hmg/ddx179 28482024

[B27] Ishihara N. Hamasaki M. Yokota S. Suzuki K. Kamada Y. Kihara A.. (2001). Autophagosome requires specific early Sec proteins for its formation and NSF/SNARE for vacuolar fusion. Mol. Biol. Cell 12, 3690–3702. 10.1091/mbc.12.11.3690 11694599 PMC60286

[B28] Jakel R. J. Maragos W. F. (2000). Neuronal cell death in Huntington’s disease: a potential role for dopamine. Trends Neurosci. 23, 239–245. 10.1016/s0166-2236(00)01568-x 10838590

[B30] Kara N. Z. Flaisher-Grinberg S. Anderson G. W. Agam G. Einat H. (2018). Mood-stabilizing effects of rapamycin and its analog temsirolimus: relevance to autophagy. Behav. Pharmacol. 29, 379–384. 10.1097/FBP.0000000000000334 28777104

[B31] Katrancha S. M. Koleske A. J. (2015). SNARE complex dysfunction: a unifying hypothesis for schizophrenia. Biol. Psychiatry 78, 356–358. 10.1016/j.biopsych.2015.07.013 26296424 PMC4703341

[B32] Kulbe J. R. Mulcahy Levy J. M. Coultrap S. J. Thorburn A. Bayer K. U. (2014). Excitotoxic glutamate insults block autophagic flux in hippocampal neurons. Brain Res. 1542, 12–19. 10.1016/j.brainres.2013.10.032 24505621 PMC3934833

[B33] Lazzeri G. Lenzi P. Busceti C. L. Ferrucci M. Falleni A. Bruno V.. (2007). Mechanisms involved in the formation of dopamine-induced intracellular bodies within striatal neurons. J. Neurochem. 101, 1414–1427. 10.1111/j.1471-4159.2006.04429.x 17286589

[B34] Lee H-J. Cho E-D. Lee K. W. Kim J-H. Cho S-G. Lee S-J. (2013). Autophagic failure promotes the exocytosis and intercellular transfer of α-synuclein. Exp. Mol. Med. 45:e22. 10.1038/emm.2013.45 23661100 PMC3674407

[B35] Lenzi P. Lazzeri G. Biagioni F. Busceti C. L. Gambardella S. Salvetti A.. (2016). The autophagoproteasome a novel cell clearing organelle in baseline and stimulated conditions. Front. Neuroanat. 10:78. 10.3389/fnana.2016.00078 27493626 PMC4955296

[B36] Limanaqi F. Gambardella S. Biagioni F. Busceti C. Fornai F. (2018). Epigenetic effects induced by methamphetamine and methamphetamine-dependent oxidative stress. Oxid. Med. Cell. Longev. 2018:28 10.1155/2018/4982453 PMC6081569 30140365

[B37] Lin M. Chandramani-Shivalingappa P. Jin H. Ghosh A. Anantharam V. Ali S.. (2012). Methamphetamine-induced neurotoxicity linked to ubiquitin-proteasome system dysfunction and autophagy-related changes that can be modulated by protein kinase C delta in dopaminergic neuronal cells. Neuroscience 210, 308–332. 10.1016/j.neuroscience.2012.03.004 22445524 PMC3358550

[B38] Linhart R. Wong S. A. Cao J. Tran M. Huynh A. Ardrey C.. (2014). Vacuolar protein sorting 35 (Vps35) rescues locomotor deficits and shortened lifespan in *Drosophila* expressing a Parkinson’s disease mutant of Leucine-Rich Repeat Kinase 2 (LRRK2). Mol. Neurodegener. 9:23. 10.1186/1750-1326-9-23 24915984 PMC4126812

[B39] Lipton J. O. Sahin M. (2014). The neurology of mTOR. Neuron 84, 275–291. 10.1016/j.neuron.2014.09.034 25374355 PMC4223653

[B40] Loder M. K. Melikian H. E. (2003). The dopamine transporter constitutively internalizes and recycles in a protein kinase C-regulated manner in stably transfected PC12 cell lines. J. Biol. Chem. 278, 22168–22174. 10.1074/jbc.M301845200 12682063 PMC2597781

[B41] Longatti A. Tooze S. A. (2012). Recycling endosomes contribute to autophagosome formation. Autophagy 8, 1682–1683. 10.4161/auto.21486 22874560 PMC3494599

[B42] Maday S. Holzbaur E. L. (2012). Autophagosome assembly and cargo capture in the distal axon. Autophagy 8, 858–860. 10.4161/auto.20055 22617438 PMC3378425

[B43] Marshansky V. Futai M. (2008). The V-type H^+^-ATPase in vesicular trafficking: targeting, regulation and function. Curr. Opin. Cell Biol. 20, 415–426. 10.1016/j.ceb.2008.03.015 18511251 PMC7111286

[B44] Masini D. Bonito-Oliva A. Bertho M. Fisone G. (2018). Inhibition of mTORC1 signaling reverts cognitive and affective deficits in a mouse model of Parkinson’s disease. Front. Neurol. 9:208. 10.3389/fneur.2018.00208 29686643 PMC5900003

[B45] Moreau K. Ravikumar B. Renna M. Puri C. Rubinsztein D. C. (2011). Autophagosome precursor maturation requires homotypic fusion. Cell 146, 303–317. 10.1016/j.cell.2011.06.023 21784250 PMC3171170

[B46] Nair U. Jotwani A. Geng J. Gammoh N. Richerson D. Yen W. L.. (2011). SNARE proteins are required for macroautophagy. Cell 146, 290–302. 10.1016/j.cell.2011.06.022 21784249 PMC3143362

[B47] Newell-Litwa K. Salazar G. Smith Y. Faundez V. (2009). Roles of BLOC-1 and adaptor protein-3 complexes in cargo sorting to synaptic vesicles. Mol. Biol. Cell 20, 1441–1453. 10.1091/mbc.E08-05-0456 19144828 PMC2649275

[B48] Nirenberg M. J. Chan J. Liu Y. Edwards R. H. Pickel V. M. (1996). Ultrastructural localization of the vesicular monoamine transporter-2 in midbrain dopaminergic neurons: potential sites for somatodendritic storage and release of dopamine. J. Neurosci. 16, 4135–4145. 10.1523/JNEUROSCI.16-13-04135.1996 8753875 PMC6579002

[B49] Okerlund N. D. Schneider K. Leal-Ortiz S. Montenegro-Venegas C. Kim S. A. Garner L. C.. (2017). Bassoon controls presynaptic autophagy through Atg5. Neuron 93, 897.e7–913.e7. 10.1016/j.neuron.2017.01.026 28231469

[B50] Palmisano N. J. Rosario N. Wysocki M. Hong M. Grant B. Meléndez A. (2017). The recycling endosome protein RAB-10 promotes autophagic flux and localization of the transmembrane protein ATG-9. Autophagy 13, 1742–1753. 10.1080/15548627.2017.1356976 28872980 PMC5640181

[B51] Parra L. A. Baust T. B. Smith A. D. Jaumotte J. D. Zigmond M. J. Torres S.. (2016). The molecular chaperone Hsc70 interacts with tyrosine hydroxylase to regulate enzyme activity and synaptic vesicle localization. J. Biol. Chem. 291, 17510–17522. 10.1074/jbc.M116.728782 27365397 PMC5016148

[B52] Pasquali L. Lazzeri G. Isidoro C. Ruggieri S. Paparelli A. Fornai F. (2008). Role of autophagy during methamphetamine neurotoxicity. Ann. N Y Acad. Sci. 1139, 191–196. 10.1196/annals.1432.016 18991864

[B53] Pavlos N. J. Gronborg M. Riedel D. Chua J. J. Boyken J. Kloepper T. H.. (2010). Quantitative analysis of synaptic vesicle rabs uncovers distinct yet overlapping roles for Rab3a and Rab27b in Ca^2+^-triggered exocytosis. J. Neurosci. 30, 13441–13453. 10.1523/JNEUROSCI.0907-10.2010 20926670 PMC6634719

[B54] Puri C. Renna M. Bento C. F. Moreau K. Rubinsztein D. C. (2013). Diverse autophagosome membrane sources coalesce in recycling endosomes. Cell 154, 1285–1299. 10.1016/j.cell.2013.08.044 24034251 PMC3791395

[B55] Ravikumar B. Moreau K. Jahreiss L. Puri C. Rubinsztein D. C. (2010). Plasma membrane contributes to the formation of pre-autophagosomal structures. Nat. Cell Biol. 12, 747–757. 10.1038/ncb2078 20639872 PMC2923063

[B56] Rizzoli S. O. (2014). Synaptic vesicle recycling: steps and principles. EMBO J. 33, 788–822. 10.1002/embj.201386357 24596248 PMC4194108

[B57] Rost B. R. Schneider F. Grauel M. K. Wozny C. Bentz C. Blessing A.. (2015). Optogenetic acidification of synaptic vesicles and lysosomes. Nat. Neurosci. 18, 1845–1852. 10.1038/nn.4161 26551543 PMC4869830

[B58] Rusten T. E. Stenmark H. (2009). How do ESCRT proteins control autophagy? J. Cell Sci. 122, 2179–2183. 10.1242/jcs.050021 19535733

[B100] Ryskalin L. Busceti C. L. Limanaqi F. Biagioni F. Gambardella S. Fornai F. (2018a). A focus on the beneficial effects of alpha synuclein and a re-appraisal of synucleinopathies. Curr. Protein Pept. Sci. 19, 598–611. 10.2174/1389203718666171117110028 29150919 PMC5925871

[B59] Ryskalin L. Limanaqi F. Frati A. Busceti C. L. Fornai F. (2018b). mTOR-related brain dysfunctions in neuropsychiatric disorders. Int. J. Mol. Sci. 19:E2226. 10.3390/ijms19082226 30061532 PMC6121884

[B60] Sakane A. Manabe S. Ishizaki H. Tanaka-Okamoto M. Kiyokage E. Toida K.. (2006). Rab3 GTPase-activating protein regulates synaptic transmission and plasticity through the inactivation of Rab3. Proc. Natl. Acad. Sci. U S A 103, 10029–10034. 10.1073/pnas.0600304103 16782817 PMC1502500

[B61] Sato S. Uchihara T. Fukuda T. Noda S. Kondo H. Saiki S.. (2018). Loss of autophagy in dopaminergic neurons causes Lewy pathology and motor dysfunction in aged mice. Sci. Rep. 8:2813. 10.1038/s41598-018-21325-w 29434298 PMC5809579

[B62] Schneider J. L. Miller A. M. Woesner M. E. (2016). Autophagy and schizophrenia: a closer look at how dysregulation of neuronal cell homeostasis influences the pathogenesis of schizophrenia. Einstein J. Biol. Med. 31, 34–39. 10.23861/EJBM201631752 28239307 PMC5321090

[B63] Sheehan P. Zhu M. Beskow A. Vollmer C. Waites C. L. (2016). Activity-dependent degradation of synaptic vesicle proteins requires Rab35 and the ESCRT pathway. J. Neurosci. 36, 8668–8686. 10.1523/JNEUROSCI.0725-16.2016 27535913 PMC4987437

[B64] Shehata M. Matsumura H. Okubo-Suzuki R. Ohkawa N. Inokuchi K. (2012). Neuronal stimulation induces autophagy in hippocampal neurons that is involved in AMPA receptor degradation after chemical long-term depression. J. Neurosci. 32, 10413–10422. 10.1523/JNEUROSCI.4533-11.2012 22836274 PMC6703735

[B65] Shen D. N. Zhang L. H. Wei E. Q. Yang Y. (2015). Autophagy in synaptic development, function, and pathology. Neurosci. Bull. 31, 416–426. 10.1007/s12264-015-1536-6 26139541 PMC5563709

[B66] Shi M. Shi C. Xu Y. (2017). Rab GTPases: the key players in the molecular pathway of Parkinson’s disease. Front. Cell. Neurosci. 11:81. 10.3389/fncel.2017.00081 28400718 PMC5369176

[B67] Sillitoe R. V. Vogel M. W. (2008). Desire, disease, and the origins of the dopaminergic system. Schizophr. Bull. 34, 212–219. 10.1093/schbul/sbm170 18283047 PMC2632401

[B68] Soukup S. F. Kuenen S. Vanhauwaert R. Manetsberger J. Hernández-Díaz S. Swerts J.. (2016). A LRRK2-dependent EndophilinA phosphoswitch is critical for macroautophagy at presynaptic terminals. Neuron 92, 829–844. 10.1016/j.neuron.2016.09.037 27720484

[B69] Spang N. Feldmann A. Huesmann H. Bekbulat F. Schmitt V. Hiebel C.. (2014). RAB3GAP1 and RAB3GAP2 modulate basal and rapamycin-induced autophagy. Autophagy 10, 2297–2309. 10.4161/15548627.2014.994359 25495476 PMC4502700

[B70] Stavoe A. K. H. Hill S. E. Hall D. H. Colón-Ramos D. A. (2016). KIF1A/UNC-104 transports ATG-9 to regulate neurodevelopment and autophagy at synapses. Dev. Cell. 38, 171–185. 10.1016/j.devcel.2016.06.012 27396362 PMC4961624

[B71] Stenmark H. (2009). Rab GTPases as coordinators of vesicle traffic. Nat. Rev. Mol. Cell Biol. 10, 513–525. 10.1038/nrm2728 19603039

[B72] Südhof T. C. (2004). The synaptic vesicle cycle. Annu. Rev. Neurosci 27, 509–547. 10.1146/annurev.neuro.26.041002.131412 15217342

[B73] Südhof T. C. (2013). Neurotransmitter release: the last millisecond in the life of a synaptic vesicle. Neuron 80, 675–690. 10.1016/j.neuron.2013.10.022 24183019 PMC3866025

[B75] Szatmári Z. Kis V. Lippai M. Hegedus K. Farago T. Lorincz P.. (2014). RAB11 facilitates cross-talk between autophagy and endosomal pathway through regulation of Hook localization. Mol. Biol. Cell 25, 522–531. 10.1091/mbc.E13-10-0574 24356450 PMC3923643

[B74] Szatmári Z. Sass M. (2014). The autophagic roles of Rab small GTPases and their upstream regulators: a review. Autophagy 10, 1154–1166. 10.4161/auto.29395 24915298 PMC4203544

[B76] Takahashi Y. Meyerkord C. L. Hori T. Runkle K. Fox T. E. Kester M.. (2011). Bif-1 regulates Atg9 trafficking by mediating the fission of Golgi membranes during autophagy. Autophagy 7, 61–73. 10.4161/auto.7.1.14015 21068542 PMC3039731

[B77] Tang F. L. Erion J. R. Tian Y. Liu W. Yin D. M. Ye J.. (2015). VPS35 in dopamine neurons is required for endosome-to-golgi retrieval of Lamp2a, a receptor of chaperone-mediated autophagy that is critical for α-synuclein degradation and prevention of pathogenesis of Parkinson’s disease. J. Neurosci. 35, 10613–10628. 10.1523/JNEUROSCI.0042-15.2015 26203154 PMC4510296

[B78] Toda H. Mochizuki H. Flores R. III. Josowitz R. Krasieva T. B. Lamorte V. J.. (2008). UNC-51/ATG1 kinase regulates axonal transport by mediating motor-cargo assembly. Genes Dev. 22, 3292–3307. 10.1101/gad.1734608 19056884 PMC2600757

[B79] Uytterhoeven V. Lauwers E. Maes I. Miskiewicz K. Melo M. N. Swerts J.. (2015). Hsc70–4 deforms membranes to promote synaptic protein turnover by endosomal microautophagy. Neuron 88, 735–748. 10.1016/j.neuron.2015.10.012 26590345

[B80] Vanderwerf S. M. Buck D. C. Wilmarth P. A. Sears L. M. David L. L. Morton D. B.. (2015). Role for Rab10 in methamphetamine-induced behavior. PLoS One 10:e0136167. 10.1371/journal.pone.0136167 26291453 PMC4546301

[B81] Vazquez-Sanchez S. Bobeldijk S. Dekker M. P. van Keimpema L. van Weering J. R. T. (2018). VPS35 depletion does not impair presynaptic structure and function. Sci. Rep. 8:2996. 10.1038/s41598-018-20448-4 29445238 PMC5812998

[B82] Vijayan V. Verstreken P. (2017). Autophagy in the presynaptic compartment in health and disease. J. Cell Biol. 216, 1895–1906. 10.1083/jcb.201611113 28515275 PMC5496617

[B83] Waites C. L. Leal-Ortiz S. A. Okerlund N. Dalke H. Fejtova A. Altrock W. D.. (2013). Bassoon and Piccolo maintain synapse integrity by regulating protein ubiquitination and degradation. EMBO J. 32, 954–969. 10.1038/emboj.2013.27 23403927 PMC3616282

[B86] Wang Y. Li L. Hou C. Lai Y. Long J. Liu J.. (2016). SNARE-mediated membrane fusion in autophagy. Semin. Cell Dev. Biol. 60, 97–104. 10.1016/j.semcdb.2016.07.009 27422330 PMC5161566

[B85] Wang T. Martin S. Papadopulos A. Harper C. B. Mavlyutov T. A. Niranjan D.. (2015). Control of autophagosome axonal retrograde flux by presynaptic activity unveiled using botulinum neurotoxin type a. J. Neurosci. 35, 6179–6194. 10.1523/JNEUROSCI.3757-14.2015 25878289 PMC4787026

[B84] Wang H. Xu J. Lazarovici P. Zheng W. (2017). Dysbindin-1 involvement in the etiology of schizophrenia. Int. J. Mol. Sci. 18:E2044. 10.3390/ijms18102044 28937620 PMC5666726

[B87] Weinstein J. J. Chohan M. O. Slifstein M. Kegeles L. S. Moore H. Abi-Dargham A. (2017). Pathway-specific dopamine abnormalities in schizophrenia. Biol. Psychiatry 81, 31–42. 10.1016/j.biopsych.2016.03.2104 27206569 PMC5177794

[B89] Wu S. Fagan R. R. Uttamapinant C. Lifshitz L. M. Fogarty K. E. Ting A. Y.. (2017). The dopamine transporter recycles via a retromer-dependent postendocytic mechanism: tracking studies using a novel fluorophore-coupling approach. J. Neurosci. 37, 9438–9452. 10.1523/JNEUROSCI.3885-16.2017 28847807 PMC5618262

[B88] Wu Q. Xu H. Wang W. Chang F. Jiang Y. Liu Y. (2016). Retrograde trafficking of VMAT2 and its role in protein stability in non-neuronal cells. J. Biomed. Res. 30, 502–509. 10.7555/JBR.30.20160061 27924069 PMC5138583

[B90] Yamamoto H. Kakuta S. Watanabe T. M. Kitamura A. Sekito T. Kondo-Kakuta C.. (2012). Atg9 vesicles are an important membrane source during early steps of autophagosome formation. J. Cell Biol. 198, 219–233. 10.1083/jcb.201202061 22826123 PMC3410421

[B91] Zavodszky E. Seaman M. N. Moreau K. Jimenez-Sanchez M. Breusegem S. Y. Harbour M. E.. (2014). Mutation in VPS35 associated with Parkinson’s disease impairsWASH complex association and inhibits autophagy. Nat. Commun. 5:3828. 10.1038/ncomms4828 24819384 PMC4024763

[B92] Zhang M. Schekman R. (2013). Cell biology. Unconventional secretion, unconventional solutions. Science 340, 559–561. 10.1126/science.1234740 23641104

[B93] Zhao J. Zhai B. Gygi S. P. Goldberg A. L. (2015). mTOR inhibition activates overall protein degradation by the ubiquitin proteasome system as well as by autophagy. Proc. Natl. Acad. Sci. U S A 112, 15790–15797. 10.1073/pnas.1521919112 26669439 PMC4703015

[B94] Zhen Y. Stenmark H. (2015). Cellular functions of Rab GTPases at a glance. J. Cell Sci. 128, 3171–3176. 10.1242/jcs.166074 26272922

